# Causes of death in Vanuatu

**DOI:** 10.1186/s12963-016-0074-4

**Published:** 2016-03-15

**Authors:** Karen Carter, Viran Tovu, Jeffrey Tila Langati, Michael Buttsworth, Lester Dingley, Andy Calo, Griffith Harrison, Chalapati Rao, Alan D. Lopez, Richard Taylor

**Affiliations:** Secretariat of the Pacific Community (SPC), B.P. D5, Nouméa, Cedex 98848 New Caledonia; School of Population Health (SPH), Faculty of Health Sciences, University of Queensland (UQ), St Lucia, Brisbane, Queensland 4072 Australia; Ministry of Health (MoH), Port Vila, Vanuatu; Formerly, Ministry of Health (MoH), Port Vila, Vanuatu; World Health Organisation, Vanuatu office, Port Vila, Vanuatu; National Statistics Office, Port Vila, Vanuatu; National Centre for Epidemiology and Population Health, Research School of Population Health, Australian National University (ANU), Acton, Canberra, Australian Capital Territory (ACT) 2601 Australia; Melbourne School of Population and Global Health, University of Melbourne, Parkville, Melbourne, Victoria 3010 Australia; School of Public Health and Community Medicine (SPHCM), University of New South Wales (UNSW), Randwick, Sydney, New South Wales (NSW) 2052 Australia

**Keywords:** Vanuatu, Cause of death, Maternal mortality, Child deaths, Adult deaths, Infection, Non-communicable disease

## Abstract

**Background:**

The population of the Pacific Melanesian country of Vanuatu was 234,000 at the 2009 census. Apart from subsistence activities, economic activity includes tourism and agriculture. Current completeness of vital registration is considered too low to be usable for national statistics; mortality and life expectancy (LE) are derived from indirect demographic estimates from censuses/surveys. Some cause of death (CoD) data are available to provide information on major causes of premature death.

**Methods:**

Deaths 2001–2007 were coded for cause (ICDv10) for ages 0–59 years from: hospital separations (HS) (*n* = 636), hospital medical certificates (MC) of death (*n* = 1,169), and monthly reports from community health facilities (CHF) (*n* = 1,212). Ill-defined causes were 3 % for hospital deaths and 20 % from CHF. Proportional mortality was calculated by cause (excluding ill-defined) and age group (0–4, 5–14 years), and also by sex for 15–59 years. From total deaths by broad age group and sex from 1999 and 2009 census analyses, community deaths were estimated by deduction of hospital deaths MC. National proportional mortality by cause was estimated by a weighted average of MC and CHF deaths.

**Results:**

National estimates indicate main causes of deaths <5 years were: perinatal disorders (45 %) and malaria, diarrhea, and pneumonia (27 %). For 15–59 years, main causes of male deaths were: circulatory disease 27 %, neoplasms 13 %, injury 13 %, liver disease 10 %, infection 10 %, diabetes 7 %, and chronic respiratory disease 7 %; and for females: neoplasms 29 %, circulatory disease 15 %, diabetes 10 %, infection 9 %, and maternal deaths 8 %. Infection included tuberculosis, malaria, and viral hepatitis. Liver disease (including hepatitis and cancer) accounted for 18 % of deaths in adult males and 9 % in females. Non-communicable disease (NCD), including circulatory disease, diabetes, neoplasm, and chronic respiratory disease, accounted for 52 % of premature deaths in adult males and 60 % in females. Injuries accounted for 13 % in adult males and 6 % in females. Maternal deaths translate into an annual maternal mortality ratio of 130/100,000 for the period.

**Conclusion:**

Vanuatu manifests a double burden of disease with significant proportional mortality from perinatal disorders and infection/pneumonia <5 years and maternal mortality, coupled with significant proportional mortality in adults (15–59 years) from cardiovascular disease (CVD), neoplasms, and diabetes.

## Background

The Southwest Pacific Melanesian country of Vanuatu gained independence in 1980, and is composed of an archipelago of mostly volcanic (“high”) islands. The population was 234,000 at the 2009 census [1], with over 100 languages represented. The archipelago is located in the Pacific’s most southwestern extent of the range of anopheline mosquitoes (the Buxton line) and thus endemic malaria. Apart from subsistence, economic activity includes tourism and agriculture.

Current reporting completeness from routine civil registration and vital statistics (CRVS) systems in Vanuatu are considered too low to enable data to be corrected and used directly to derive estimates of mortality level [1]. Consequently, measures of levels of mortality and LE are derived from demographic estimates from censuses and surveys. Data on mortality level were produced from the Vanuatu Multiple Indicator Cluster Survey (MICS) which in 2007 reported on under-5 year mortality (U5M) and the infant mortality rate (IMR) [2], and Vanuatu has also recently completed a Demographic and Health Survey (DHS) [3]. Demographic analyses of censuses have also produced information on level of mortality using questions on Children Ever Born, Children Surviving (CEBCS) for <5 years mortality, with or without adult mortality estimates from parental orphanhood methods to impute model life tables [1, 4] (1989 [5], 1979 [6]). However, neither these surveys nor censuses have collected information on CoD. Empirical data for circa 2010 reports an IMR of around 20/1,000 and life expectancies of 68 years for males and 72 years for females, although these figures may underestimate IMR and overestimate LE [7].

World Health Organization (WHO) reports estimate proportional mortality by CoD for Vanuatu “based on a combination of country life tables, regional cause of death patterns, and WHO and UNAIDS program estimates for some major causes of death” and “have a high degree of uncertainty” [8]. Similarly, Global Burden of Disease (GBD) estimates are also based on expected patterns of CoD derived from estimated level of mortality and other parameters [9].

However, a variety of CoD data are available from hospital and community deaths in Vanuatu, and these can provide useful information on major causes of premature mortality and hence important public health issues in Vanuatu. Available CoD information from Hospital Separations (HS), medical certification of hospital deaths (MC), and Community Health Facilities (CHF) sources for 2000–2007 is used to generate proportional mortality for CoD by age and sex. Similarities and differences in the resultant CoD distributions for these sources are reviewed, and source-weighted national averages (by age/sex) are estimated to assess the contribution of these disease categories to premature mortality, and to assist with identification of key public health issues contributing to premature mortality in Vanuatu.

## Methods

### Data sources

De-identified unit record data on deaths for 2000–2007 were obtained during in-country visits from hospital sources with HS (as death) and MC of death (issued at hospitals), and notices of death submitted with monthly nursing reports from CHF below hospital level – area health centers, dispensaries, and aid posts. Certification and reporting of deaths in Vanuatu are discussed in detail in On et al. 2009 [10]. Because of de-identification, the two sources of hospital deaths (HS, MC) were not linkable. Deaths with no age (*n* = 138) and fetal deaths (*n* = 248) were excluded from all data sources, and hospital deaths (*n* = 21) were removed from community health facility data (Table 1). Only deaths 0–59 years were coded for cause because data quality of CoD data was less for those ≥60 years, LE in Vanuatu is likely to be above 60 years, and this age range includes the demographic adult age bracket 15–59 years. Ill-defined causes were 2.5–3.5 % in hospital deaths and 19.5 % in CHF deaths for ages 0–59 years.

**Table 1:** Cause of death review, Vanuatu 2000–2007

Causes of death by age	Hospital deaths				Community health facility deaths n (%)		National weighted proportional mortality
	Hospital separations n (%)		Medical certificates n (%)				
0–4 years	147 (16 %)		391 (24 %)		401 (17 %)		18 %
Specific causes	145		383		311		
Ill-defined %	2 (1.4)		8 (0.6)		90 (22.4)		
5–14 years	35 (4 %)		37 (2 %)		73 (3 %)		2 %
Specific causes	32		36		55		
Ill-defined %	3 (8.6)		1 (2.7)		18 (24.7)		
15–59 years	M	F	M	F	M	F	
	228	226	440	301	409	329	
Specific causes	224	219	418	291	335	275	
Ill-defined %	4 (1.8)	7 (3.1)	22 (5.0)	10 (3.3)	74 (18.1)	54 (16.4)	
Both sexes 15–59 years	454 (49 %)		741 (46 %)		738 (31 %)		35 %
0–59 years with cause	636 (69 %)		1169 (73 %)		1212 (51 %)		
Specific causes	620		1128		976		
Ill defined %	16 (2.5)		41 (3.5)		236 (19.5)		
≥60 years^a^	291(31 %)		443 (28 %)		1182 (49 %)		45 %
All ages deaths 0 - ≥60 years	927 (100 %)		1612 (100 %)		2394 (100 %)		100 %
Deaths excluded from above							
Fetal deaths	13		100		102		
No age	14		78		46		
Hospital deaths	-		-		21		
All reported deaths							
Reported deaths	954		1790		2563		
% deaths with cause 0–59 years	97.2		90.1		93.4		
% of all Vanuatu deaths^b^	9.6		17.3		25.2		

Vanuatu national proportional mortality weighted by data sources (MC and CHF) for age groups

^a^Not coded for cause of death. *M* Male; *F* Female

^b^Estimated total deaths from available life tables (excluding fetal deaths)

### Hospital deaths

For **HS**, CoD data were collected from the two main hospitals: the national referral hospital at Port Vila on Efate (the main Island), and the secondary district hospital at Luganville, the main town on Espirito Santo (*n* = 954), excluding 27 deaths (no age recorded, and still births) (Table 1). These are the larger two of only three hospitals in Vanuatu routinely staffed by doctors. Hospital separation data were extracted from the Health Information System (HIS). This database is maintained at the national level, but local versions of the database were used as they are considered to be more complete. CoD is recorded according to principal diagnosis, with up to two additional fields for other causes as well as fields for accidents, external causes, and medical procedures. Hospital separation data would normally have the principal diagnosis recorded first, but this field was recorded as the mode of death (cardio-respiratory arrest, heart stopped, stopped breathing, etc.) in many of the cases reviewed, indicating that tabulation based on the first field of the database would have been misleading. The fields appear to have been completed as a causal sequence, as found on a medical certificate, so underlying cause was selected and coded according to ICDv10 rules for medical certificates [11]. Causes were coded by medical record staff at the hospital, with both text and code entered in the database. **Medical certificates** for hospital deaths are completed semi-routinely (and particularly upon request by family members). During the period of this study, medical certificates were not routinely coded or collated for Luganville, and although collated for Port Vila hospital, were not routinely coded, analyzed, or reported. Data were extracted from the original medical certificates at Luganville hospital and from a spreadsheet maintained by medical records staff at Port Vila hospital (*n* = 1,790). The spreadsheet included all causes listed in Part I of the certificate as text, identified by line. Contributory causes were not recorded. Medical certificates are predominantly completed by the attending doctor. Although almost all certified deaths occurred in hospital, doctors occasionally certified community deaths. Place of death was not well-recorded. Underlying cause was selected and coded (by KC) according to ICDv10 [11] (Table 1).

### Community health facility deaths

Deaths in the community are captured through health system reporting by health centers, dispensaries, and aid posts. The locally trained nurses or nurse aides at each facility are required to submit a monthly report of activities including any deaths in their area. A notice of death form is also collected for deaths in the community. This includes name, age, date of death, place of death, and CoD as a single free-text field. Deaths are collated and coded according to ICDv10 and entered into the national HIS database. Data were sorted according to health facility code, and deaths that occurred in Vila or Luganville hospitals were removed. For deaths where specific age was not recorded, deaths recorded as “adults” were attributed to age group 15–59 years, and “old” or “elderly” to age group ≥60 years. There were 2,563 deaths recorded by CHF between 2001 and 2007, with an average of 366 deaths per year, or approximately 26 % of the expected deaths in Vanuatu based on available life tables [12]. For deaths reported from Port Vila hospital, 21 were removed from the CHF data set; all were adult deaths (13 aged 15–59 years, and eight aged ≥60 years).

### Analysis

The approximate proportion of total deaths accounted for by each data set was calculated based on an average of 1,394 deaths per year as estimated by the Vanuatu National Statistics Office [12], assuming little variation over the period under investigation.

Fetal deaths were identified and removed from all data sets: 13 from hospital medical certificates, 100 from hospital separation data, and 102 from community health facility data. Cases where there was uncertainty whether the death was fetal or early neonatal were counted as neonatal, as separate reporting mechanisms exist for stillbirths; in practice they should not be recorded in these data. Deaths without ages were also removed: 14 deaths from medical certificates, 78 from hospital deaths, and 46 deaths from facility data (Table 1).

Deaths were tabulated by broad age group: 0–4 years and 5–14 years for both sexes (because of small numbers), 15–59 years by sex to evaluate differences in CoD distribution by age, and ≥ 60 years (not coded for CoD). Causes of death were aggregated to ICDv10 General Mortality List One (104 causes), with childhood deaths (0–4 years) aggregated to ICDv10 Condensed List Three for U5M. Tabulations of reported deaths by cause and proportional mortality, excluding unknown and ill-defined deaths, were then calculated for each data source. Exclusion of unknown/ill-defined deaths from analysis of proportional mortality produces the same result as redistribution of deaths by cause.

In order to produce national estimates of proportional mortality by cause, estimated total deaths by broad age group (<5, 5–14, 15–59, ≥60 years) and sex were derived from census analyses by application of reported annual age- and sex-specific mortality rates to populations (from the 1999 census) [4] and estimated annual deaths by age/sex reported in the 2009 census analytic report [1]. These data were averaged to obtain estimated annual Vanuatu deaths by age/sex for the study period. Average annual community deaths were estimated from total deaths by deduction of annualized MC deaths as the best representation of hospital deaths. This produced source-weights for <5 years of 0.33 for MC and 0.67 for CHF deaths, and for 5–14 years of 0.15 for MC and 0.85 for CHF deaths (both sexes); and for 15–59 years 0.32 for MC and 0.68 for CHF male deaths, and 0.37 for MC and 0.63 for CHF female deaths. National proportional mortality by cause was estimated by a weighted average of MC and CHF proportional mortality. The national estimate of proportional mortality from maternal mortality was used to estimate maternal deaths from the adult female population, and this was divided by births derived from the 1999 and 2009 census analyses [1, 4] to produce a maternal mortality ratio for the period.

## Results

For 2000–2007, there were 954 HS as death (from the two major hospitals) and 1,790 MC (almost all hospital deaths) available for analysis, before exclusions; the age distributions of deaths were similar between the two overlapping hospital sources (Table 1). Deaths reported from CHF (*n* = 2,563) were characterized by a lesser proportion of adult death (15–59 years) and a higher proportion of elderly deaths compared to the hospital sources. Only deaths <60 years were coded for CoD. The proportion of ill-defined causes was <5 % for hospital deaths, but for CHF, ill-defined deaths accounted for 23 % of deaths <15 years and 17 % in adults (15–59 years). Further, deaths from specific causes in adults, not included in the major causes of death, were 5–10 % for hospital deaths and 22–25 % for community deaths (Table 1).

For females, main causes were neoplasms 29 %, circulatory disease 15 %, diabetes 10 %, infection 9 %, and maternal deaths 8 %. Infection included tuberculosis (3 % for both sexes), malaria, and viral hepatitis. Liver disease (including hepatitis and cancer) accounted for 18 % of deaths in adult males and 9 % in females. Circulatory disease and diabetes caused 34 % of adult deaths in males and 25 % in females; with neoplasm and chronic respiratory disease, NCD accounted nationally for 52 % of premature deaths in males and 60 % in females. External causes (injuries) were responsible for 13 % in adult males and 6 % in females. Maternal deaths translate into a maternal mortality ratio of 130/100,000 for the period.

For children <5 years the main CoD were perinatal disorders in all sources of data. Infectious diseases, including diarrhea, malaria, and respiratory disease, especially pneumonia, were more prominent in CHF deaths than in hospital deaths. National estimates for proportional <5 years mortality were 45 % perinatal disorders and 27 % infection/pneumonia (Table 2).

**Table 2:** Cause of death in children (both sexes), Vanuatu 2000–2007

Cause of death by age group	Hospital deaths				Community health facility deaths		National weighted^a^ proportional mortality %
	Hospital separations		Medical certificates				
	No.	%	No.	%	No.	%	
Age 0–4 years							
Infection, parasitic, including	18	12.4	21	5.5	58	18.6	14.2
*Diarrhea*	*4*	*2.8*	*8*	*2.1*	*23*	*7.4*	5.6
*Malaria*	*7*	*4.8*	*5*	*1.3*	*29*	*9.3*	6.6
Endocrine, metabolic, nutrition	4	2.8	12	3.1	14	4.5	4.0
Nervous system	7	4.8	5	1.3	7	2.3	2.0
Respiratory system, including	14	9.7	31	8.1	63	20.3	16.2
*Pneumonia*	*10*	*6.9*	*17*	*4.4*	*51*	*16.4*	12.4
Perinatal disorders, including	71	49.0	232	60.6	115	37.0	44.9
*Complications of pregnancy, childbirth*	*3*	*2.1*	*41*	*10.7*	*40*	*12.9*	12.2
*Low birth weight*	*34*	*23.4*	*84*	*21.9*	*24*	*7.7*	12.4
*Intra-uterine hypoxia, birth asphyxia*	*16*	*11.0*	*55*	*14.4*	*5*	*1.6*	5.9
Congenital disorders	13	9.0	41	10.7	19	6.1	7.6
All other diseases	2	4.8	3	5.7	2	5.5	5.6
External causes	7	2.8	22	3.1	17	4.5	4.0
Total specific cause 0–4 years	145	100.0	383	100.0	311	100.0	100.0
*Ill-defined excluded*	*2*	*1.4*	*8*	*2.0*	*90*	*22.4*	15.6
Age 5–14 years							
Infection, parasitic, including	8	22.9	8	22.2	13	23.6	23.4
*Malaria*	*4*	*11.4*	*5*	*13.9*	*10*	*18.2*	17.5
Neoplasms	4	11.4	3	8.3	6	10.9	10.5
Blood	1	2.9	2	5.4	3	4.1	4.3
Endocrine, metabolic, nutrition	4	11.4	0	0.0	1	1.8	1.5
Nervous system, including	4	11.4	7	19.4	5	9.1	10.7
*Meningitis*	*3*	*8.6*	*6*	*16.7*	*1*	*1.8*	4.1
Circulatory system	5	14.3	1	2.8	6	10.9	9.7
Respiratory system	1	2.9	2	5.6	4	7.3	7.0
Digestive system	0	0.0	1	2.8	4	7.3	6.6
Congenital disorders	3	8.6	1	2.8	0	0.0	0.4
All other diseases	1	2.9	4	11.1	0	0.0	1.7
External causes	1	2.9	7	19.4	13	23.6	23.0
Total specific cause 5–14 years	35	100.0	36	100.0	55	100.0	100.0
*Ill-defined excluded*	*3*	*8.6*	*1*	*2.7*	*18*	*24.7*	21.3

Cause of death according to ICDv10 General Mortality List 1 for Chapters and causes of interest with sufficient deaths. Proportional mortality by cause calculated excluding (1–094) ill-defined and unknown. Hospital deaths: Hospital Separations (HS) as deceased. Medical Certificate (MC) of death. Community Health Facility (CHF): reporting of deaths (excluding hospitals).

^a^Vanuatu national proportional mortality by cause weighted by data sources (MC and CHF) for age groups

For children 5–14 years (only 3–4 % of deaths by source), the most important cause of death in all data sources was infection at 33–45 % of deaths including malaria, meningitis, and respiratory deaths. External causes (injuries) were prominent in hospital medical certificates (19 %) and CHF (24 %) data (Table 2).

For adults (15–59 years), circulatory disease was the most common in males at 27–29 % (by source), and neoplasms were the most common in females at 28–32 % (Table 3). The next most common causes of death in males, after circulatory disease, were: neoplasms (12–16 %), external causes (injuries) (9–13 %), then endocrine, metabolic, nutritional, etc. diseases (mostly diabetes) at 6–10 %. National proportional mortality (weighted) for males was: circulatory disease 27 %, neoplasms 13 %, injury 13 %, liver disease 10 %, infection 10 %, diabetes 7 %, and chronic respiratory disease 7 %. For females, the next most common causes of adult deaths after neoplasms were circulatory disease (14–16 %), then endocrine, metabolic, nutritional, etc. (mostly diabetes) at 10–13 %. National proportional mortality (weighted) for females was: neoplasms 29 %, circulatory disease 15 %, diabetes 10 %, infection 9 %, and maternal deaths 8 %.

**Table 3:** Adult proportional mortality by cause 15–59 years, Vanuatu 2000-2007

Cause of death	Males							Females						
	Reported deaths			Proportional mortality (%)				Reported deaths			Proportional mortality (%)			
	Hospital		CHF	Hospital		CHF		Hospital		CHF	Hospital		CHF	
	HS	MC		HS	MC		Van^b^	HS	MC		HS	MC		Van^b^
Infection	21	41	32	9.3	9.8	9.6	9.7	22	27	23	10.1	9.1	8.4	8.7
Respiratory TB	3	4	9	1.3	1.0	2.7	2.2	1	5	8	0.5	1.7	2.9	2.5
Other TB	1	3	1	0.4	0.7	0.3	0.4	4	3	-	1.8	1.0	-	0.4
Viral hep	6	20	7	2.7	4.8	2.1	3.0	4	4	5	1.8	1.4	1.8	1.7
AIDS	-	1	-	-	0.2	-	0.1	-	-	-	-	-	-	-
Malaria	4	2	10	1.8	0.5	3.0	2.2	4	5	7	1.8	1.7	2.5	2.2
Neoplasms	35	48	47	15.5	11.5	14.1	13.3	66	91	77	30.3	31.5	28.0	29.3
Liver etc	10	20	14	4.5	4.8	4.2	4.4	5	3	8	2.3	1.0	2.9	2.2
Breast	-	1	-	-	0.2	-	-	9	27	10	4.1	9.4	3.6	5.7
Cervix uteri	-	-	-	-	-	-	-	13	20	7	5.9	7.0	2.5	4.2
Endocrine etc.	16	41	19	7.1	9.9	5.7	7.0	29	28	31	13.3	9.8	11.3	10.7
Diabetes	16	40	18	7.1	9.7	5.4	6.8	28	28	29	12.8	9.8	10.5	10.2
Nervous system	5	11	16	2.2	2.6	4.8	4.1	3	6	8	1.4	2.0	2.9	2.6
Circulatory dis	64	119	89	28.5	28.8	26.6	27.3	34	46	39	15.5	16.0	14.2	14.9
Rheumatic	3	7	-	1.3	1.7	-	-	7	13	-	3.2	4.5	-	-
Hypertensive	7	5	2	3.1	1.2	0.6	0.8	2	2	4	0.9	0.7	1.5	1.2
IHD	15	51	2	6.7	12.3	0.6	4.3	4	7	-	1.8	2.4	-	-
Other heart	26	33	71	11.6	8	21.2	17.0	9	10	24	4.1	3.5	8.7	6.8
Stroke	13	21	14	5.8	5.1	4.2	4.5	12	12	11	5.5	4.2	4.0	4.1
Respiratory dis	19	34	24	8.5	8.2	7.2	7.5	16	18	19	7.3	6.2	7.0	6.7
COPD	10	21	18	4.5	5.1	5.4	5.3	9	13	14	4.1	4.5	5.1	4.9
Alimentary dis	23	33	46	10.3	7.9	13.7	11.9	10	15	19	4.6	5.2	6.9	6.3
Liver disease	21	29	40	9.4	7.0	11.9	10.4	7	10	16	3.2	3.5	5.8	5.0
Genito-urinary	14	21	10	6.3	5.1	3.0	3.7	23	21	11	10.5	7.3	4.0	5.2
Maternal	-	-	-	-	-	-	-	7	13	28	3.2	4.5	10.2	8.1
External	20	52	42	8.8	12.6	12.6	12.6	3	17	15	1.4	5.8	5.6	5.7
MVA	9	8	3	4.0	1.9	0.9	1.2	-	1	-	-	0.3	-	-
Drowning	3	9	9	1.3	2.2	2.7	2.5	-	-	3	-	-	1.1	0.7
Assault	1	8	7	0.4	1.9	2.1	2.0	2	4	5	0.9	1.4	1.8	1.7
Other causes	11	40	84	4.9	9.6	25.1	20.2	13	19	59	5.9	6.5	21.5	16.0
All specific cause	224	418	335					219	291	275				
Ill-defined^a^ excluded	4	22	74	1.8	5.0	18.1		7	10	54	3.1	3.3	16.4	

Cause of death according to ICDv10 Chapters and causes of interest with sufficient deaths; *COPD* Chronic Obstructive Pulmonary Disease

*HS* Hospital separations as deceased; *MC* Medical certificate of hospital death; *CHF* Community Health Facility (excluding hospitals) deaths

^a^Proportional mortality by cause calculated excluding (1–094) ill-defined and unknown

^b^Vanuatu national proportional mortality by cause weighted by data sources (MC and CHF) for age group by sex

Infection in adults included tuberculosis (3 % for both sexes), malaria, and viral hepatitis. Liver disease (including hepatitis and cancer) accounted for 18 % of deaths in adult males and 9 % in females. Circulatory disease and diabetes caused 34 % of adult deaths in males and 25 % in females; with neoplasm and chronic respiratory disease, NCD accounted nationally for 52 % of premature adult deaths in males and 60 % in females. External causes (injuries) were responsible for 13 % in adult males and 6 % in females.

Maternal deaths were responsible for 10 % of adult female deaths in CHF, and 3–5 % in hospital data sources, with a weighted national average of 8 %. This translates to approximately 8.2 deaths per year for the country for the period under study, which produces a maternal mortality ratio of 130 per 100,000 births (from the 1999 and 2009 censuses).

## Discussion

This study reports empirical cause of death patterns from Vanuatu based on age 0–59 years from hospital and community health facility data sources. Deaths in the HS data overlap those from MC on deaths in hospital and provide two similar perspectives on cause of death patterns from hospital sources. The age distributions of death (all ages) and proportional mortality for the main causes in adults by age group (0–59 years) for hospital deaths are quite similar, providing some cross validation of representativeness and validity of the two hospital sources. However, the MC data may be reliable and valid due to larger coverage for the same period and certification by a medical doctor, even though it contains a few community deaths for which a medical certificate of death was sought. For all deaths from CHF, proportional distribution of deaths by age was lower for age 15–59 years (31 %) compared to hospital sources (46–49 %), and higher for age ≥60 years (49 %) compared to hospital sources (28–31 %). For age 0–4 years, proportional mortality from infectious diseases was higher from CHF (19 %) than hospital sources (6–12 %), as was respiratory disease (20 %) compared to hospital sources (8–10 %). In adults aged 15–59 years, proportional mortality was remarkably similar for major causes of death from CHF compared to hospital sources, except for maternal deaths which were 10 % in CHF compared to 3–5 % from hospital sources. In 0–59 year olds, ill-defined causes were evident in 20 % of CHF, whereas for hospital sources the proportion was 3–4 %. Deaths with ill-defined cause were excluded from calculation of proportion mortality from specific causes. Deaths from specific causes in adults, not included in the major causes of death, were 22–25 % for community deaths and 5–10 % for hospital deaths. Certification and reporting of deaths in Vanuatu are discussed in detail in On et al. 2009 [10].

For deaths among those aged 0–4, all sources of data reflect the importance of neonatal causes of death, demonstrating the importance of antenatal care, appropriate delivery services, improved maternal health, and good quality neonatal care [13, 14] in reducing mortality in this age group. Infectious diseases (pneumonia, malaria, meningitis, and diarrhea) represent a significant proportion of deaths in this age group, as reported by all sources. The 2013 DHS indicated that for children <5 years in the two weeks prior to the survey: 3 % had symptoms of acute respiratory infection, 13 % of children had fever, and 12 % had diarrhea [3]. At the DHS survey, 28 % showed evidence of stunting, 4 % wasting, 11 % underweight, and 2 % were obese for their age [3]. This highlights the importance of programs such as the Integrated Management of Childhood Illnesses (IMCI) programs [15], to target these areas at a community level to accelerate progress towards reducing infant and childhood mortality under the post-2015 Sustainable Development Goals (SDGs), having failed to reach the Millennium Development Goals (MDGs) for these indices. For 5-14-year-olds, small numbers preclude detailed analysis, but the most common CoD were infection-related. External causes were also prominent in some data sources.

The leading causes of death for adults aged 15–59 were circulatory disease, neoplasms, diabetes, and liver diseases. Differences in cause distribution between “other heart diseases” and specific diagnoses (e.g., ischemic heart disease and cerebrovascular disease) between sources in this age group possibly reflect both more accurate reporting by doctors on the medical certificates and greater likelihood of these deaths being recorded as non-specific heart disease in community health facility reports by nurses and nursing aides – rather than reflecting a true difference in underlying disease patterns. For adult females, cervical and breast cancer rank highly as CoD in both the hospital and health facility data. NCDs were the leading causes of premature death for adults in both sexes in the 15–59 age group. For males, these were predominantly CVD and diabetes, while for females, neoplasms were the most important CoD followed by CVD and diabetes.

Compared to hospital sources, deaths in CHF had higher proportions of elderly (≥60 years) deaths coded as ill-defined (20 %) for 0–59 years, and higher CoD proportions due to infectious and respiratory diseases in children <5 years. This indicates the importance of CHF data for providing information on out-of-hospital deaths, particularly for children and the elderly, and the need to improve diagnostic specificity for CHF deaths.

The GBD 2010 estimates rank CoD for Vanuatu as: stroke (7.4 %), lower respiratory disease (6.9 %), ischemic heart disease (6.3 %), diabetes (5.5 %), preterm birth complications (4.3 %), diarrheal disease (3.2 %), tuberculosis (2.7 %), and road injury (2.5 %), measured as proportion of total years of life lost (YLL) [9]. Thus around 20 % of YLL are due to CVD or diabetes.

Although it is not possible to provide national estimates of cause-specific mortality rates per population due to the uncertainty in existing data collections (both for level and cause of mortality), the data presented in this paper clearly demonstrate Vanuatu is dealing with a double burden of disease, with significant proportions of mortality attributed to infectious diseases, perinatal, and maternal mortality for Group I, and non-communicable disease (NCD) as Group II conditions [16]. While the significant proportion of NCDs in adults from CHF data may, in part, be attributed to reporting of non-specific causes such as “heart problem” and “heart-stopped,” this pattern is also reflected in the hospital deaths and adds to the evidence that the mortality burden from NCDs is significant. The significant proportion of deaths attributed to NCDs in adults aged 15–59 years, including CVD and diabetes, is consistent with patterns seen in other Pacific states such as Nauru [17], Tonga [18, 19], and Fiji [20, 21], where NCDs have contributed to elevated premature adult mortality and thus limited improvements in LE [7].

The significant proportion of “other cancers” reported in Vanuatu adults is attributable, at least in part, to a prominence of thyroid cancer which is consistent with published incidence data for Vanuatu, and is not dissimilar to several other Pacific Island populations [22, 23]. Previous studies report a higher incidence in Vanuatu of cervical cancer and high-grade epithelial abnormalities (2 %) on screening than Australia [24], which is consistent with findings that show cervical cancer as an important CoD in adult females. Screening is, however, very limited, and human papillomavirus (HPV) vaccine, which would benefit future generations of women, is not yet generally available.

The deaths from liver disease and liver cancer are most likely associated with viral hepatitis, although CoD is not available at this level. Hepatitis B antigen carriage in adults ≥20 years was reported as 15 % in 1989 [25] and 25 % in adult women (mothers) in 1991 [26]. Successful immunization for hepatitis B in ni-Vanuatu children since the 1990s [27, 28] should lead to fewer cases of liver disease in adults in the next generation.

Deaths from external causes appear underrepresented in the hospital separation data (but not the MC or CHF data), possibly because they died at the scene or were dead on arrival (and hence not admitted to hospital), and as such may not have been adequately considered in public health priorities.

Maternal deaths were 10 % of adult female deaths 15–59 years in CHF and 4–5 % in hospital data, or 8 % as a weighted national estimate. This translates into a maternal mortality ratio of 130/100,000 births for 2000–2009. The hospital maternal mortality ratio was reported as 70/10^5^ births from the labor ward at Port Vila hospital for 1979–2001 [29]. Modelled estimates of maternal mortality in Vanuatu are reported as 230/100,000 in 2000 and 178 in 2008 [30]. The Vanuatu MICS in 2007 [2] reported that 24 % of women were married before 18 years of age, 92 % of urban births were in health facilities and 78 % in rural areas, and 87 % of urban births were delivered by skilled personnel, while in rural areas it was 72 %. The 2009 census reported that the adolescent (15–19 years) fertility rate was higher in rural (77/1000) than urban areas (40/1000) [1]. These data are congruent with the higher proportional maternal mortality from community health facility deaths compared to hospital deaths in Vanuatu.

Despite data shortcomings, findings presented here provide national-level CoD data that can assist health planners and donor agencies ensure that health assessment and intervention programs are more accurately targeted towards diseases and conditions responsible for the greatest proportions of premature mortality. Furthermore, this analysis highlights the strengths and weaknesses of the various data sets available, and indicates how they how they should be improved and could be used in future analyses.

## Conclusion

Vanuatu manifests a double burden of disease with significant proportional mortality from perinatal disorders and infection/pneumonia <5 years and maternal mortality, indicative of a pre-transitional pattern, coupled with significant proportional mortality in adults aged 15–59 years from CVD, neoplasms, and diabetes suggestive of later stages in the epidemiological transition.

## Abbreviations

ADRAS: Australian development research award
ANU: Australian National University
CEBCS: children ever-born and children surviving
CHF: community health facilities
CoD: cause of death
CRVS: civil registration and vital statistics
CVD: cardiovascular disease
DFAT: department of foreign affairs and trade
DHS: demographic and health survey
HIS: health Information system
HPV: human papillomavirus
HS: hospital separations
IHME: institute for health metrics and evaluation
IMCI: integrated management of childhood illnesses
IMR: infant mortality rate
LE: life expectancy
MDG: millennium development goal
MICS: multiple indicator cluster survey
MoH: vanuatu ministry of health
MS: medical certificates
NCD: non-communicable disease
SPC: secretariat of the pacific community
SPH: school of population health
SPHCM: school of public health and community medicine
U5M: under-5 mortality
VNSO: vanuatu national statistics office
WHO: World Health Organization
YLL: years of life lost
